# The aqueous extract of *Olea europaea* leaves protects from haematotoxicity and kidney damage induced by diclofenac in Swiss albino mice

**DOI:** 10.1039/c9ra01670h

**Published:** 2019-07-29

**Authors:** Raouya Soussi, Najla Hfaiedh, Mohsen Sakly, Khémais Ben Rhouma

**Affiliations:** Laboratory of Integrated Physiology, Faculty of Science of Bizerte, University of Carthage Tunisia 7021 Jarzouna Bizerte Tunisia raouiya_soussi@yahoo.fr +21622507475; Research Unit of Macromolecular Biochemistry and Genetics, Faculty of Sciences of Gafsa, University of Gafsa Gafsa 2112 Tunisia

## Abstract

*Olea europaea* leaves are one of the most widely used by-products in traditional medicine due to their biological properties. This study evaluated the antioxidant activities, and the beneficial effects of the aqueous extract of “Sahli” *Olea europaea* leaves on diclofenac-induced haematotoxicity and nephrotoxicity in Swiss albino mice. The mice were divided into four groups of seven each: a control group, a diclofenac-treated group, a group orally gavaged with the extract of olive leaves, and a group pre-treated with the extract of olive leaves and then injected with diclofenac. The results obtained indicated that the injection of the mice with diclofenac alone caused an extensive change in their haematological and biochemical parameters, such as red and white blood cells (RBC and WBC, respectively), platelet count (PLT), and creatinine and urea levels, a significant increase in lipid peroxidation level (TBARS) and a decrease in superoxide dismutase (SOD), catalase (CAT), and glutathione peroxidase (GPX) levels. Olive leaf extract administration in the diclofenac-treated mice was found to correct and restore all the investigated parameters and protect the kidney histology by minimizing the oxidative stress induced by diclofenac in the mice tissues.

## Abstract 2

*Olea europaea* leaves are one of the most widely used by-products in traditional medicine due to their biological properties.

## Graphical abstract

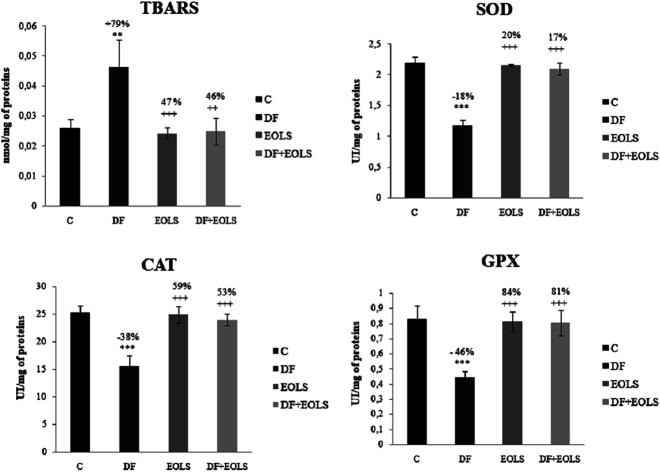

## Introduction

Non-steroidal anti-inflammatory drugs (NSAID) are a diverse group of chemical agents^1^ with similar biological capabilities, regularly used for pain relief and the treatment of many inflammatory diseases.^2^ Diclofenac (DF) belongs to the family of NSAID, which is considered as one of the most commonly used painkillers.^3^

This product has some potent biological activities that affect cellular function in every organ,^4^ and it has also been shown that the efficacy of DF in reducing pain and inflammation is associated with various side reactions mostly affecting the gastrointestinal system, kidneys and risk of heart attack.^5^ Thus, nephrotoxicity is one of the most common kidney problems, which occurs when the body is exposed to a drug or toxin.^6^ In addition, the long-term therapeutic consumption of DF is reportedly associated with some adverse effects on haematological, biochemical and oxidative parameters as well as chronic nephrotoxicity.^2,9^

Currently, the use of herbal medicines continues to expand rapidly worldwide^8^ because of their minimal side effects as compared to synthetic drugs,^9^ which are regarded as unsafe to humans and the environment.^9^

A large number of medicinal plants have been used for thousands of years in the traditional system of medicine.^10^ Among them, olive tree is considered as a source of bioactive compounds^11^ and has been known since ancient times for its medicinal properties.^12^

In Tunisia, olives (*Olea europaea*, *Oe*) are abundantly found in more than 50 different cultivars.^13^ Several studies have reported that there is an increasing interest in olive by-products and, in particular, olive leaves.^14^

Olive leaves have always aroused significant interest due to their various bioactivities and traditional therapies.^15^ It has been demonstrated that olive leaf extract has significant antioxidant and antimicrobial activities due to its richness in phenolic components, which can be considered as a source of potential antioxidant and antimicrobial agents.^16^

Therefore, the aim herein was to evaluate the protective effects of the extract of “Sahli” (EOLS) olive leaves on the haematological and biochemical parameters, kidney histopathological changes and the implication of oxidative stress on this organ in mice inflicted by diclofenac. Accordingly, the haematological parameters and renal biomarkers of mice treated with diclofenac were assessed. In addition, the status of the oxidative stress was determined by measuring the activity of antioxidant enzymes such as superoxide dismutase (SOD), catalase (CAT), and glutathione peroxidase (GPX), and the lipid peroxidation level (TBARS), and thereafter, the histopathological changes in the kidneys of the mice were examined.

Furthermore, a phytochemical analysis (DPPH and FRAP) was conducted to determine the antioxidant properties of the plant extract, and then the HPLC analysis was performed, which revealed the presence of phenolic and flavonoids compounds.

## Material and methods

### Chemicals

DF, a non-steroidal anti-inflammatory drug (NSAID) that belongs to the acetic acid group, was purchased from the Central Pharmacy of Gafsa Tunisia, which was produced by the “DAR ESSAYDALI LABORATORY” Tunisia to be dissolved in saline solution for injection.

### Plant and sample preparation

Fresh leaves of the cultivar “Sahli” *Olea europaea* leaves were collected at the beginning of October 2014 from the Gafsa area located in South Western Tunisia (longitude: 8.47E, latitude: 34.255N, altitude: 295 m, rainfall: 160 mm per year) (*T* °C min: 3.9 January, *T* °C max: 38.4 August) with the help of a botanist at the Biological Sciences Department, Faculty of Sciences of Gafsa.

After collection, the plant leaves were washed with distilled water to eliminate any traces of dust, and subsequently dried in the shade for 15 days, then ground to a fine powder and packaged in plastic bags and stored at 4 °C for further analysis.

### *Olea europaea* leaf extract preparation

The air-dried plant material was extracted by maceration. 250 mL of boiling water was added to the olive leaf powder (5 g) for 24 h with continuous stirring. Subsequently, the solution was filtered, and finally the extract was stored in the dark at room temperature for cooling, and then it was used for the *in vivo* treatment.

### Experimental design

#### Animals

A total of twenty-eight Swiss albino male mice weighing approximately 25–30 g, were obtained from the Central Pharmacy (SIPHAT, Tunisia). The experimental protocol was approved by the Ethical Committee of the Faculty of Sciences of Bizerte.

Animal maintenance and experimental procedures were carried out in accordance with the International Guidelines for Care and Use of Laboratory Animals of Tunis University. The animals were maintained for a two-week adaptation period under standard conditions of temperature of 22 ± 2 °C, relative humidity of 50 ± 4%, and a constant photoperiod 12 h/dark cycle. Animals were fed with 15% protein food pellets obtained from the “Société Industrielle de Conditionnement Optimisé” (S.I.C.O.) Sfax, Tunisia and had tap water *ad libitum*.

The mice were treated according to the Tunisian Code of Practice for the Care and Use of Animals for Scientific Purposes and the European Convention for the Protection of Vertebrate Animals used for Experimental and other Scientific Purposes (Council of Europe no. 123, Strasbourg, 1985).

### Experimental procedure

The mice were randomly divided into four experimental groups of approximately similar weight (*n* = 6) as follows:

Group 1 (C): mice served as the control group drinking water *ad libitum* and received a standard laboratory diet for 28 days.

Group 2 (DF): mice received diclofenac by intraperitoneal injection at a dose of 2.37 mg kg^−1^ body weight for 5 days following the protocol of Thanagari *et al.* (2012).^3^

Group 3 (EOLS): mice were given EOLS at a dose of 3.3 g kg^−1^ of body weight by daily oral gavage for 28 days.^12^

Group 4 (EOLS + DF): mice were pre-treated with EOLS by oral gavage at a dose of 3.3 g kg^−1^ for 23 days and then injected with diclofenac for 5 days at the end of the experimental period.

At the end of all treatments, the animals from each group were rapidly sacrificed by decapitation to avoid the effect of stress. Blood samples were collected from their jugular vein, centrifuged (1500 × *g*, 15 min, 4 °C) and immediately used for analysis of haematological parameters and biochemical assays. The kidneys were removed, cleaned from fat and stored at −80 °C until use.

#### Preparation of kidney extracts

About 1 g of kidney was cut into small pieces, then homogenized in 2 mL of Tris buffered-saline solution (TBS) (pH 7.4) using a crusher (Ultra-Turrax grinder). Subsequently, the homogenate was centrifuged (5000 g, 30 min) (4 °C), and finally the supernatants were recovered and stored at −80 °C until use.

### Biochemical assays

#### Evaluation of lipid peroxidation

The level of lipid peroxidation was measured as thiobarbituric acid reactive substances (TBARS) according to Yagi *et al.* (1976).^17^ The test involved the combination of 125 μL of supernatant (S1) of kidney homogenate and 175 μL of 20% trichloroacetic acid containing 1% butyl-hydroxytoluene and centrifugation (1000 × *g*, 10 min, 4 °C). Thereafter, 200 μL of supernatant (S2) was mixed with 40 μL of HCL (0.6 M) and 160 μL of thiobarbituric acid (0.72 mM) and the mixture was heated at −80 °C for 10 min, and then its absorbance was measured at 530 nm. The total TBARS amount was calculated using an extinction coefficient of 156 mM^−1^ cm^−1^ and expressed as nmol mg^−1^ protein.

#### Antioxidant enzymes assay

The activities of antioxidant enzymes, SOD, CAT, and GPX were measured in kidney homogenates of all the experimental animals. The total (Cu, Zn and Mn) SOD activity was determined according to the method of Durak *et al.* (1993).^18^ This activity was determined by measuring the ability to inhibit the photo-reduction of nitroblue tetrazolium (NBT). One unit of SOD represents the amount inhibiting the photo-reduction of NBT by 50%. The activity was expressed as units per mg protein at 25 °C.

Catalase (CAT) activity was measured according to the method by Aebi *et al.* (1984).^19^

The reaction mixture (1 mL) contained 100 mM phosphate buffer solution (pH 7), 100 mM H_2_O_2_ and 20 μL (about 1–1.5 mg of protein) of the kidney homogenate. Hydrogen peroxide (H_2_O_2_) decomposition was determined at 25 °C by the decrease in absorbance at 240 nm for 1 min. Enzyme activity was calculated using the extinction coefficient of 0.043 mM^−1^ cm^−1^ and expressed in international units (IU), *i.e.* in mmol H_2_O_2_ destroyed min^−1^ mg^−1^ protein. Glutathione peroxidase (GPX) activity was assayed using the method described by Flohe and Gunzler (1984)^20^ at 25 °C and was expressed as mmol of GSH oxidized min^−1^ g^−1^ protein.

#### Determination of haematological parameters

Heparin was added to the blood samples used for the biochemical assays. Red blood cells (RBC), white blood cells (WBC), platelets count (PLT), haemoglobin (Hb), hematocrit (Ht), mean corpuscular volume (VCM) and mean cell haemoglobin concentration (MCHC) levels from EDTA (ethylenediaminetetraacetic acid) tubes were analysed using an electronic automatic apparatus (MAXM, Beckman Coulter Inc., Fullerton, USA).

#### Plasma marker assays

Plasma was collected by centrifugation (1500 × *g*, 15 min, 4 °C) and the levels of creatinine and urea were determined using commercial kits (Spinreact).

#### Histopathological study of the kidney

Thin slices of the mouse kidney were fixed for 48 h at room temperature by direct immersion in a fixative solution (4% formaldehyde in 0.1 M phosphate buffer, pH 7.4). The samples were dehydrated with ethanol, cleaned with toluene and embedded in paraffin. The paraffin sections were cut into 5–6 μm thick slices and then stained with hematoxylin and eosin (H&E) and examined under a light microscope.

#### Experimental conditions for high-performance liquid chromatography (HPLC)

HPLC analysis was carried out with a Varian ProStar model 230 (Varian Associates, Walnut Creek, CA) analytical HPLC system equipped with a ternary pump (model Q2 Prostar 230) and a photodiode array detector (model Prostar 335). HPLC separation of the active compounds was conducted on a 5 μm particle C-18 reversed-phase column (Zorbax, 250 mm × 4.6 mm). The mobile phase was composed of solvent A: methanol 100% and solvent B: 0.05% acetic acid aqueous solution. The gradient elution used was 75% A and 25% B; 30 min = 60% A and 40% B; 40 min = 90% A and 10% B. The flow rate was 1 mL min^−1^ and the injection volume was 20 μL at 25 °C.^21^ Identification of the compounds was carried out by comparing the retention time and mass spectra of the peaks in the injected sample extracts to that of HPLC standard compounds.

#### Determination of total phenolic content

The total phenolic content of the *Olea europaea* leaf extract was determined according to the method of Folin *et al.* (1927)^22^ using gallic acid as a standard, and the absorbance was measured at 765 nm. Total phenolic content was expressed as mg gallic acid equivalent (GAE) g^−1^ extract.

#### Determination of total flavonoid content

The total flavonoid content was determined according to the method described by Zhishen *et al.* (1999),^23^ where quercetin was used as the standard, and the absorbance was measured at 510 nm. The flavonoid content was expressed as mg of equivalent of rutin per g of dry matter.

### Antioxidant assay

#### DPPH radical scavenging activity

The free radical scavenging activity of the *Oe* leaf extract was evaluated using the DPPH radical assay according to the method reported by Grzegorczyk *et al.* (2007).^24^ The sample was diluted in methanol at different concentrations (50–500 μg mL^−1^). Then 1 mL of each diluted plant extract was added to 0.1 mM DPPH methanolic solution. The mixtures with different sample concentrations and DPPH were placed in the dark at room temperature for 30 min. This was followed by measurement of the absorbance at 517 nm. The antiradical activity was expressed as IC_50_ (μg mL^−1^).

DPPH radical scavenging (RSA) expressed as a percentage was calculated using the following formula:RSA% = [*A*_DPPH_(*A*_sample_ − *A*_control_)] × 100/*A*_DPPH_where, *A*_DPPH_ is the absorbance in the presence of DPPH, *A*_sample_ is the absorbance in the presence of the extract, and *A*_control_ is the absorbance of the control.

#### Ferric reducing antioxidant power (FRAP)

The ability of the *Oe* extracts to reduce Fe^3+^ was assayed by the method of Oyaizu (1986).^25^

1 mL of different sample concentrations (20–100 μg mL^−1^) was mixed with 2.5 mL of potassium phosphate buffer (0.1 mol L^−1^, pH = 6.6) and 2.5 mL of 1% potassium ferricyanide (K_3_Fe(CN)_6_). After incubation in a water bath at 50 °C for 20 min, 2.5 mL of 10% trichloroacetic acid was added to the mixture and centrifuged at 3000 rpm for 10 min. Finally, the supernatant (2.5 mL) was mixed with 2.5 mL distilled water and 0.5 mL of 0.1% ferric chloride solution (FeCl_3_). The mixture was incubated at 28 °C for 30 min to facilitate a colour change, and the intensity of the blue-green colour was measured at 700 nm as a function of *Oe* leaf extract concentration in mg mL^−1^ and then compared with that of ascorbic acid, which was used as the standard. The increase in the absorbance of the reaction indicated an increase in the reducing power of the extracts.

#### Statistical analysis

All tests were performed in triplicate and the results are expressed as mean ± standard deviation (SD). The determination of all parameters was performed from six animals per group. Statistical significance was determined using one-way ANOVA, followed by Tukey's *post hoc* test. *P* < 0.05 was considered statistically significant.

## Results

### Haematological parameters

Exposure of the mice to DF for five consecutive days caused a significant reduction in red blood cells (RBC), white blood cells (WBC), haemoglobin (Hg), haematocrit (Ht), mean corpuscular volume (VCM) and mean cell haemoglobin concentration (MCHC) values (5.03 ± 0.78 × 10^6^/μL, 5.71 ± 1.50 × 10^3^/μL, 9.73 ± 0.82 g dL^−1^, 30.66 ± 2.1%, 36.3 ± 7.4 10^−6^ μm^3^ per RBC, 28.10 ± 0.84 g dL^−1^, respectively), while a significant increase in platelet number (887.33 ± 7.73 × 10^3^ μL) as compared to the control group (C) was observed (Table 1).

**Table 1:** Haematological parameters in the control, diclofenac-treated, olive leaf Sahli extract-treated and olive leaf Sahli-diclofenac-treated mice^a^

Parameter	C	DF	EOLS	EOLS + DF
RBC (10^6^/μL)	7.83 ± 0.14	5.03 ± 0.78**	7.61 ± 0.41^++^	7.25 ± 0.52^++^
WBC (10^3^/μL)	10.55 ± 0.25	5.71 ± 1.50**	10.12 ± 0.39^++^	10.22 ± 0.41^++^
Hb (g dL^−1^)	12.63 ± 0.90	9.73 ± 0.82**	11.27 ± 0.92^+^	11.49 ± 0.87^+^
PLT (10^3^/μL)	610 ± 5.19	887.33 ± 7.73**	609.3 ± 9.6^+++^	668.6 ± 10.25^++^
HT (%)	43.32 ± 1.26	30.66 ± 2.1***	43.1 ± 1.47^+^	39.23 ± 0.68^++^
VCM (10^−6^ μm^3^ per RBC)	50.76 ± 1.64	36.3 ± 7.4 *	49.86 ± 2.08^+^	50.53 ± 1.17^+++^
MCHC (g dL^−1^)	32.33 ± 0.49	28.10 ± 0.84**	32.7 ± 0.65^++^	30.04 ± 0.605^+^

a C: control group, DF: diclofenac-treated mice, EOLS: extract of olive leaves Sahli given mice, EOLS + DF: extract of olive leaves Sahli and diclofenac-treated mice, RBC: red blood cells, WBC: white blood cells, Hb: haemoglobin, PLT: blood platelets, HT: haematocrit, VCM: mean corpuscular volume, and MCHC: mean cell haemoglobin concentration. All values are expressed as mean ± SEM. *n* = 6 for each treatment group. **p ≤ 0.01, ***p ≤ 0.001 significantly different from C group; ^+^*p* ≤ 0.05, ^++^*p* ≤ 0.01, and ^+++^*p* ≤ 0.001 significantly different from DF group.

In contrast, in the (DF + EOLS) group, these parameters were restored to normal levels (7.25 ± 0.52 × 10^6^/μL, 10.22 ± 0.41 10^3^ μL, 11.49 ± 0.87 g dL^−1^, 39.23 ± 0.68%, 50.53 ± 1.17 10^−6^ μm^3^ per RBC, 30.04 ± 0.605 g dL^−1^, 668.6 ± 10.25 × 10^3^/μL, respectively) (Table 1). Furthermore, no significant differences were observed between the (DF + EOLS)-treated mice and the control (C) group mice.

### Plasma markers of kidney damage

DF treatment induced severe kidney damage, as shown in Table 2, where the levels of creatinine and urea were significantly higher in the diclofenac-treated group (DF) (44.29 ± 1.32 μmol L^−1^ and 9.26 ± 0.92 mmol L^−1^, respectively) than in the control mice group (C). When the diclofenac-treated mice were previously treated with the *Oe* leaf extract, all these biomarkers were maintained at almost normal values.

**Table 2:** Effects of the aqueous extract of *Olea europaea* leaves (EOLS) on the creatinine (μmol L^−1^) and urea (mmol L^−1^) levels in mice with diclofenac-induced nephrotoxicity^a^

Parameter	C	DF	EOLS	EOLS + DF
Creatinine (μmol L^−1^)	34.96 ± 1.07	44.29 ± 1.32***	35.46 ± 0.50^+++^	35.96 ± 0.8^+++^
Urea (mmol L^−1^)	4.84 ± 0.37	9.26 ± 0.92**	4.3 ± 0.3^+++^	6.34 ± 0.76^++^

a C: control group, DF: diclofenac-treated mice, EOLS: extract of olive leaves Sahli given mice, EOLS + DF: extract of olive leaves Sahli and diclofenac-treated mice. All values are expressed as mean ± SEM. *n* = 6 for each treatment group. *p ≤ 0.05, **p ≤ 0.01, ***p ≤ 0.001 significantly different from control group; ^+^*p* ≤ 0.05, ^++^*p* ≤ 0.01, and ^+++^*p* ≤ 0.001 significantly different from diclofenac-treated (DF) group.

### Evaluation of the antioxidant enzymes activities and lipid peroxidation in the kidney

**Fig. 1:**
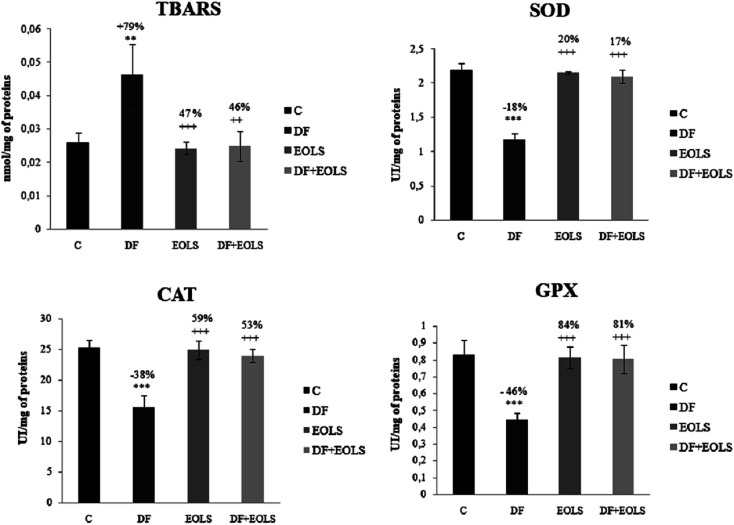
Effects of diclofenac (DF), extract of olive leaves Sahli (EOLS), and their combination (EOLS + DF) on the levels of lipid peroxidation (expressed as TBARS, nmol mg^−1^ of protein) and activities (IU per mg of protein) of superoxide dismutase (SOD), glutathione peroxidase (GPX) and catalase (CAT) in the kidney. Values were expressed as mean ± SEM of 6 mice in each group, **P ≤ 0.01 compared to control group (C) ***p ≤ 0.001 compared to control group (C); ^++^*p* ≤ 0.01 compared to diclofenac-treated group (DF), and ^+++^*p* ≤ 0.001 compared to diclofenac-treated group (DF). %: percentage of increase or decrease of TBARS and the antioxidant activity parameters (SOD, CAT, and GPX) compared to the control or diclofenac-treated group.

The effect of diclofenac administration and pre-treatment with the *Oe* leaf extract on lipid peroxidation and antioxidant enzymes in the kidney is shown in Fig. 1. Our results revealed a significant increase in TBARS content by 79% in the diclofenac-treated mice (DF) compared to the controls. The administration of the *Oe* leaf extract significantly reduced the TBARS level to the control values in the DF + EOLS group. However, treatment with EOLS alone did not significantly cause any changes compared to the control group.

The antioxidant enzyme activity, *i.e.*, SOD, CAT, and GPX, which protect against oxidative stresses, was found to decrease by −18%, −38%, and −46%, respectively, in the kidneys of the DF-treated mice compared to the controls (Fig. 1), which indicate the failing defense against oxidative stress was significantly corrected in the animals pre-treated with the olive leaf extract. However, no changes were observed in the group treated with the leaf extract only compared with the control group.

### Histopathological examination

**Fig. 2:**
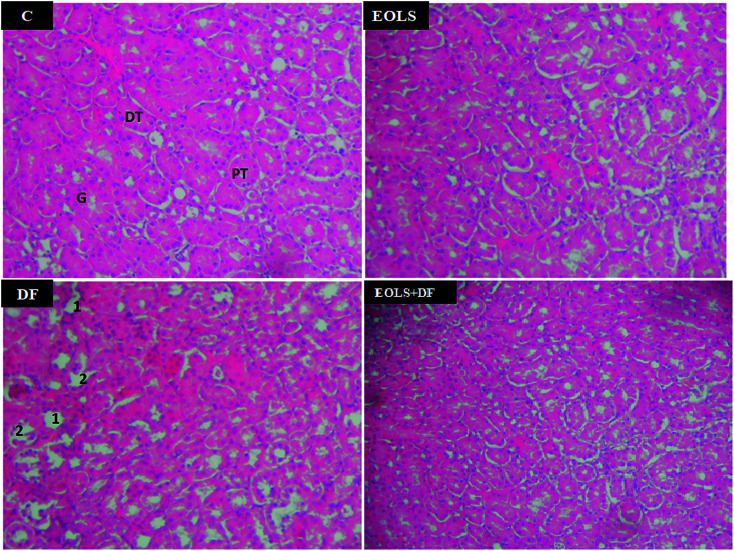
Photomicrographs of the mice kidney sections (hematoxylin and eosin, 400×): (C) control group showing the normal parenchymal architecture; (EOLS): extract of olive leaves Sahli-treated group showing normal cellular morphology similar to (C); (DF) diclofenac-treated group showing severe degenerative changes in their proximal and distal tubules, and damaged glomeruli; and (EOLS + DF) group showing a marked improvement in the histological picture. G: glomeruli, PT: proximal tubule, DT: distal tubule, 1: tubular degenerations, and 2: damaged glomeruli.

Fig. 2 shows the histopathological examination of the kidney sections of the experimental animals. The kidney section in the control (C) and EOLS-treated mice kidneys revealed normal morphology of the renal parenchyma with well-defined glomeruli and tubules. In contrast, the kidneys of the DF-treated group had a lot pathological alterations revealed by alterations and degenerative changes in the distal and proximal tubules. However, these alterations were attenuated in the case of the group pre-treated with the *Oe* leaf extract (EOLS + DF), and their the kidney sections showed the normal tubular structure compared with the control mice (C).

### HPLC analysis

**Fig. 3:**
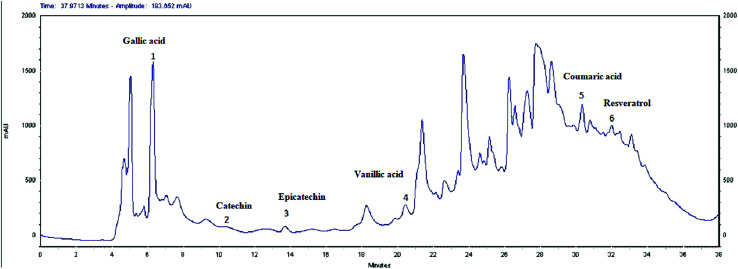
HPLC chromatogram of phenolic acids (*λ* = 280 nm) from the extract of olive leaves Sahli (EOLS), peaks: (1) gallic acid, (2) catechin, (3) epicatechin, (4) vanillic acid, (5) coumaric acid, and (6) resveratrol.

**Fig. 4:**
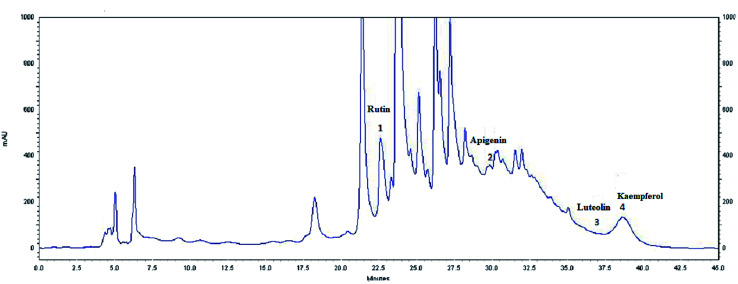
HPLC profile of flavonoids (*λ* = 360 nm) from the extract of olive leaves Sahli (EOLS), peaks: (1) rutin, (2) apigenin, (3) luteolin, and (4) kaempferol.

The HPLC analysis of EOLS revealed the presence of phenolic acids and flavonoids, and the identified bioactive compounds are summarized in Fig. 3 and 4. There were 6 known phenolic acids identified at 280 nm in the leaf extract: gallic acid, catechin, epicatechin, vanillic acid, coumaric acid, and resveratrol, with a retention time of 6.283, 9.495, 13.727, 20.585, 30.457, and 32.485 min, respectively (Fig. 3).

The HPLC elution profile of the flavonoids displayed in Fig. 4 revealed 4 compounds identified at 360 nm: rutin, apigenin, luteolin, and kaempferol, with a retention time of 22.69, 32.182, 36.205, and 41.21 min, respectively. Thus, *Oe* leaves were proven to be rich in antioxidant compounds.

### Total polyphenol and flavonoid content

The total phenolic and flavonoid content of the *Oe* leaf extract were examined and presented in Table 3, where the total phenolic content of the extract was 83.7162 ± 5.21 mg GAE g^−1^ DR, and its level was expressed as gallic acid equivalent per gram of extract. Total flavonoid was expressed as quercetin equivalent per gram of extract, which was estimated to be 41.6355 ± 5.37 mg QE g^−1^ DR.

**Table 3:** Total phenolic and flavonoid contents of *Olea europaea* leaf extract^a^

	Total phenolic acids	Total flavonoids
*Olea europaea* leaf extract	83.7162 ± 5.21 mg GAE g^−1^ DR	41.6355 ± 5.37 mg QE g^−1^ DR

a mg GAE g^−1^ DR: mg gallic acid equivalents per gram dry residue. mg QE g^−1^ DR: mg of quercetin equivalent per gram dry residue.

### Antioxidant capacities of EOLS

#### DPPH radical-scavenging activity

**Fig. 5:**
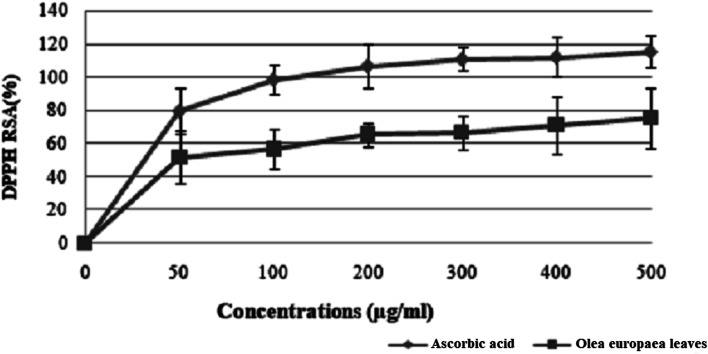
Free radical scavenging activity of *Olea europaea* leaf extract. Each value is the mean of 3 separate assays ± SD. RSA: radical scavenging activity.

The antiradical effect of the olive leaf sample was measured by DPPH radical scavenging activity. It is clear from Fig. 5 that the DPPH scavenging activity of EOLS increased in a concentration-dependent manner at a concentration of 500 μg mL^−1^, where EOLS showed a high radical scavenging activity value of 88%, but lower than that of ascorbic acid, which amounted to 118%.

The results suggest that the inhibition concentration (IC_50_) of the olive leaf extract that yielded 50% inhibition of the DPPH radical was 50 ± 1.2 μg mL^−1^, which was lower than that of ascorbic acid (35 ± 0.086 μg mL^−1^).

#### Reducing power assay

**Fig. 6:**
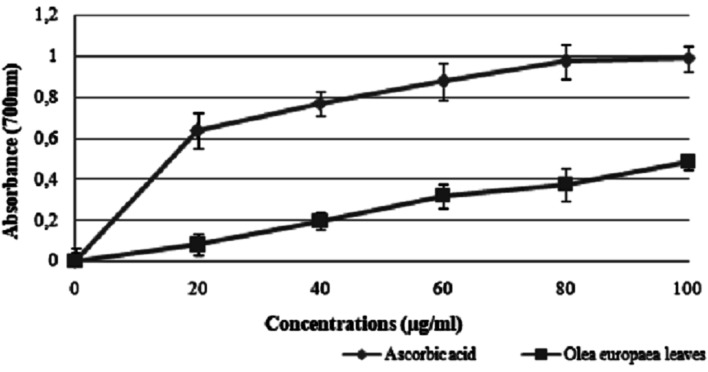
Reducing power of *Olea europaea* leaf extract and the synthetic antioxidant ascorbic acid at different concentrations.

Fig. 6 shows the ability of EOLS to reduce Fe^3+^ to Fe^2+^ at different concentrations. The reducing power of the plant leaves increased with an increase in concentration, and it was found that the reducing potential of EOLS at 100 μg mL^−1^ was 0.5 ± 0.086 μg mL^−1^, and thus lower than that of ascorbic acid, which was used as positive control (1 ± 0.063 μg mL^−1^) at the same concentration.

## Discussion

The present study was conducted to evaluate the antioxidant potency of *Oe* leaf extract *in vitro*, and to examine its protective effect against DF-induced nephrotoxicity and haematotoxicity. NSAID are regularly used as initial therapy for degenerative rheumatic diseases and painful conditions due to inflammation.^7^ Diclofenac is one of the NSAID that is widely known for its anti-inflammatory and analgesic properties.^26^ However, when it is administrated at a high dose, it has life-threating side effects.

Several studies reported that the long-term use of this drug leads to renal dysfunction,^26^ and causes changes in the haematological and biochemical parameters.^3^ Additionally, various researchers suggested that the highly damaging effect of DF on kidney tissues can possibly be the cause of acute renal failure.^27^ In fact, the kidney seems to be an early target and it is highly susceptible to toxicants.^6,27^ Actually, our study demonstrated that the administration of DF at 2.37 mg kg^−1^ dose (group DF) to mice caused significant damage in their blood parameters and kidney functions; however, the significant alterations in the haematological parameters of the mice treated with DF may provide evidence of toxicity.

In the current, the study blood analysis revealed a decrease in the levels of (RBC), (WBC), (Hb), and (HT), while the PLT counts significantly increased after exposure to diclofenac, where all these changes may suggest drug-induced toxicity. Additionally, the DF-treated mice showed damage, causing alterations in their VCM and MCHC values, resulting in anaemia, which may refer to the loss of blood. Further, the decrease in blood parameters caused by this drug may be attributed to its harmful effects on bone marrow and haematopoietic organs. Ahmad *et al.* 2013,^28^ also observed that DF decreased the heart rate, and accordingly, the oxygen carrying capacity of the blood decreased and as a result the RBC, WBC, Hb, and HT levels decreased. Additionally, our findings are in accordance with the studies by Basavraj *et al.* 2012,^29^ who also reported that DF administration in Swiss albino mice induced a significant decrease in their blood parameters.

Similar results were speculated by Shridar and Narayanan *et al.* (2007),^30^ who demonstrated that poisoning with DF induced a significant decrease in haemoglobin content and MCHC levels, and this attenuation is related to the decrease in RBC count, which may be due to the adverse effect of DF on haematological parameters. A decrease in platelet count was also reported following the same treatment, which was explained by the damages affecting the haematology function and the immune system.

On the other hand, our results showed that (DF) induced significant elevation in the serum levels of urea and creatinine compared to the control mice group (C), and the elevated serum levels of these parameters are possible indicators of kidney poisoning induced by DF. These pathological changes can be attributed to the impairment of the glomerular function and tubular damage.^30^ They can be also related to the damages affecting the structural integrity of the nephron, which is consistent with previous reports, confirming that the administration of DF leads to damage in both the proximal and distal kidney tubulars and possibly be the cause of acute renal failure.^27^

Urea and creatinine are excreted exclusively through the kidney; however, damage to the kidney will make it inefficient to excrete both urea and creatinine, resulting in their accumulation in the blood.^32^ Our study is consistent with previous reports showing an important increase in serum creatinine and urea levels in mice exposed to diclofenac.^26^

The kidneys play an important role in the elimination of toxic xenobiotics, and thus they are more likely to be exposed to toxic materials compared to other organs.^33^ Thus, in this study, the increased serum creatinine and urea levels reflect the diagnosis of renal failure.

In addition, intoxication of mice with DF created a state of oxidative stress, as indicated by an increase in lipid peroxidation level and weakening of the antioxidative status in the kidney tissue and SOD, CAT, and GPX by −18%, −38%, and −46%, respectively, compared to the control group.

SOD, CAT, and GPX play an essential role in the cellular defense, and the alteration of the antioxidant defense mechanism in mice renal tissue in response to diclofenac toxicity may be due to the excessive formation of reactive oxygen species (ROS), which develop oxidative stress in the kidneys. Thus, elevated levels of ROS due to insufficiency of the antioxidant defense system may lead to disruption of cellular function and oxidative damage to membranes.

Therefore, we conclude that oxidative stress and overproduction of ROS constitute a part of the mechanism of DF toxicity. Our results further confirmed that in earlier studies indicating that diclofenac administration induced pro-oxidative damage in renal tissue, leading to alterations in antioxidant enzymes and cell damage in kidney tissue.^34,35^

In the current study, the histological examination of the kidney sections of mice exposed to 2.37 mg kg^−1^ of DF demonstrated massive injury, where the primary victims of this drug were the distal and proximal tubules, in which we observed a marked degeneration of the architecture of the cells.

In the previous study by Hickey *et al.* (2001),^1^ they indicated that DF caused cell damage in kidney tissue^27^ and suggested that the more damaging effect of DF was on both the proximal and distal kidney tubular cells. Also, the histopathological changes in the present study confirmed the biochemical results.

Currently, herbal products symbolize safety in contrast to synthetic drugs, which are regarded as unsafe to humans and the environment.^36^ It has been reported that the natural antioxidants present in herbs are responsible for preventing the harmful consequence of oxidative stress. Accordingly, in our study, pre-treatment with the aqueous extract of *Olea europaea* leaves (EOLS) was found to significantly protect the mice from DF-induced nephrotoxicity and haematotoxicity. *Olea europaea*, a characteristic Mediterranean species, is one of the oldest agricultural tree crops worldwide,^37^ and the beneficial properties of olive leaf preparations seem to be due to its antioxidant constituents.^38^ This ameliorating effect was shown by the increase in the levels of RBC, WBC, Hb, HT, VCM, and MCHC compared to the DF-treated group. Similar results were observed by El Sayed *et al.* (2014),^39^ who demonstrated the effectiveness of pre-treatment with olive leaf extract in improving the haematological parameters towards the normal values. Additionally, in earlier studies, it was suggested that^40^ pre-treatment with olive leaves improved the haematological values including RBC, WBC, Hb, and MCHC towards the normal values. EOLS has the ability to protect blood from vanadate toxicity and stabilize damaged red blood cell membranes. Indeed, the olive leaf extract demonstrated beneficial effects on the kidney function parameters (creatinine and urea) in the present study and other works through its powerful antioxidant properties.^41^

In fact, the animals pre-treated with the olive leaf extract prior to diclofenac injection had serum creatinine and urea concentrations significantly lower than the mice treated with diclofenac alone. Considering this, we conclude that olive leaves are rich in polyphenols and their extract can be used to protect against DF nephrotoxicity.

Our results are consistent with previous reports showing that administration of EOLS had good protective effects on renal function parameters.^42^ We also found an improvement in the kidney tissues. Thus, the treatment with olive leaf extract significantly attenuated the histopathological alterations induced by DF. In agreement, our results showed that the histopathological kidney section of the mice treated with EOLS had an improved nephrocellular architecture, indicating its protective effect.

These findings are consistent with that of Al Attar and Abu Zeid (2013),^43^ who indicated that the extract of olive leaves can be considered a promising therapeutic agent against nephrotoxicity induced by toxicant agents. On the other hand, the administration of EOLS had a potent protective effect on the oxidative stress induced by DF in the kidneys of the mice. Indeed, the intoxication of mice with diclofenac created a state of oxidative stress by an increase in lipid peroxidation level and a weakening of the antioxidative status in their kidney tissue.

Actually, the aqueous *Oe* leaf extract reduced lipid peroxidation and enhanced the expression of intracellular endogenous antioxidants such us SOD, CAT and GPX by maintaining their activities at a higher level than the DF-treated mice, which is due to the ability of these enzymes to scavenge free radicals, as confirmed by Cai *et al.* (2014).^44^ It is clear that the treatment by EOLS exerted a strong protective effect. Regarding the effect of olive leaf extract administration on the oxidative responses in the DF-treated mice, it may be assumed that this extract minimised the oxidative stress induced by DF. The obtained results are consistent with that by Jafaripour *et al.* (2016),^42^ who reported that olive leaf extract exhibits important antioxidant properties.

Moreover, a phytochemical analysis of EOLS showed the presence of phenolic and flavonoid component at different relative concentrations. Specifically, the HPLC analysis showed that EOLS is rich in phenolic acids (gallic acid, catechin, epicatechin, vanillic acid, coumaric acid, and resveratrol) and flavonoids (rutin, apigenin, luteolin, and kaempferol), and similar results were also observed by Brahmi *et al.* (2012).^36^

The abundance of polyphenols and flavonoids in the aqueous extract of *Oe* may be responsible for its nephroprotective and haematoprotective efficacy since these components play an important role in absorbing and decomposing free radicals. Based on the above findings, *Oe* leaves seem to be attractive as in important source of antioxidants for the pharmaceutical industry.

In 2015, the results obtained by I. Hamad^45^ showed that methanolic extract of olive leaves cultivated in Saudi Arabia has a significant content of phenolic components and exhibit an excellent antioxidant effect on liver damages induced by CCl_4_ administration.

The use of olive leaf extract reduced the structural changes in the kidney tissue due to its antioxidant characteristics.^42^ In our experiment, the DPPH scavenging capacity was expressed as an IC_50_ value, which is the concentration of the sample required to scavenge 50% of the free radicals present in the test solution. The IC_50_ was found to be 50 ± 1.2 μg mL^−1^,^46^ which was confirmed by the findings reported in previous studies.^47^ Moreover, the results indicated that the addition of olive leaves “Sahli” led to the reduction of Fe^3+^ to Fe^2+^ by donating an electron; however, the reducing power was found to be 0.5 ± 0.086 μg mL^−1^ compared with ascorbic acid (lower than the standard antioxidant ascorbic acid). Many authors have demonstrated a linear correlation between the level of total phenolic compounds and antioxidant activities.^46^

Thereby, it was clear from our results that the improvement in the altered haematological parameters, antioxidant enzyme status and peroxidative damage in renal tissue by *Olea europaea* leaf extract can be attributed to its richness in active antioxidant compounds.

## Conclusion

In the present work, our results revealed that the aqueous extract of *Olea europaea* leaves was found to exhibit strong antioxidant activities based on numerous *in vitro* and *in vivo* assays.

However, due to its richness in polyphenol and flavonoid components, pre-treatment with EOLS in DF-injected mice improved their haematological and biochemical parameters, as well as the histopathological changes in their kidney tissues, and minimised the adverse effect of oxidative stress induced by this drug in mice tissues.

## Conflicts of interest

The authors declare that there is no conflict to interest.

## Supplementary Material
